# Exploring the acceptability and attitude toward mobile health applications to aid self-management for people with type 2 diabetes mellitus in Saudi Arabia: A descriptive cross-sectional study

**DOI:** 10.1371/journal.pone.0331314

**Published:** 2025-11-26

**Authors:** Amani Khardali

**Affiliations:** 1 Department of Clinical Practice, College of Pharmacy, Jazan University, Jizan, Jazan, Saudi Arabia; 2 Pharmacy Practice Research Unit, College of Pharmacy, Jazan University, Jizan, Jazan, Saudi Arabia; RAK Medical and Health Sciences University: Ras Al Khaimah Medical and Health Sciences University, UNITED ARAB EMIRATES

## Abstract

**Background:**

The prevalence of Type II diabetes mellitus (T2DM) is increasing, leading to a need for more sustainable and patient-centered solutions. The marketplace for mobile health (mHealth) applications (apps) that aid self-management of chronic diseases, such as T2DM, is growing. However, attitudes toward and acceptance of such technology in Saudi Arabia remain limited, particularly regarding preferences and needs. Therefore, understanding the acceptance and attitude toward using mHealth apps and preferred features is essential for planning appropriate methods and strategies to encourage usage among people with T2DM. This study aimed to determine the acceptance of and attitudes toward mHealth apps for self-management among people with T2DM in Saudi Arabia. It also seeks to understand the features that people with T2DM need and prefer regarding the mHealth apps.

**Methods:**

A cross-sectional study using a structured self-administered questionnaire was conducted on people with T2DM living in Saudi Arabia. Data were analyzed using descriptive and inferential statistics to examine acceptance, perspectives, and attitudes toward using mHealth apps.

**Results:**

A total of 424 individuals with T2DM completed the study questionnaire; among them, 92.5% owned smartphones, and 12.3% had previously used mHealth apps. Almost all respondents (98.1%) expressed willingness to use apps for self-managing T2DM. Medication reminders (78.3%) and diet management (69.8%) were the highest-ranked features of the mHealth app for T2DM self-management. Additionally, willingness to use apps for T2DM self-management was positively associated with education level and the number of medications taken daily (P = 0.002 and P = 0.04, respectively).

**Conclusion:**

Overall, using a mHealth app appears to be an acceptable approach to aid the self-management of T2DM in Saudi Arabia, especially among the younger population. The features most required for future mHealth apps were identified, which could aid the design, acceptability, and usage of such apps.

## Introduction

Diabetes mellitus (DM) is one of the most common public health concerns that can lead to several serious and long-term complications, including macrovascular and microvascular complications such as cardiovascular, renal, and eye diseases, and nerve damage [1]. The prevalence of DM in Saudi Arabia is increasing rapidly. According to the International Diabetes Federation (IDF), the prevalence was 18.7% in 2021, and it is estimated to rise to 21.4 by 2045, which would add further economic burden to healthcare institutions [2]. Type 2 Diabetes Mellitus (T2DM) is the primary driver of this increase, accounting for approximately 95% of all diabetes cases [2]. T2DM is a debilitating and multifactorial disease that may negatively impact the quality of life of people with T2DM [3]. Various management strategies should be applied to achieve therapeutic goals in T2DM, including lifestyle changes, physical activity, treatment follow-up, blood glucose control, and self-management [1].

Self-management is essential for controlling T2DM and improving patient outcomes. This includes encouraging and motivating patients to take control of their T2DM, for example, by managing their symptoms, changing their lifestyle, and becoming involved in their treatment with the support of their family, community, and healthcare providers [4,5]. Currently, several self-management tools are available worldwide, including digital technologies [3]. Digital technologies, such as smartphones for mobile health (mHealth) applications (apps) and smartwatches, are designed to help manage chronic diseases such as T2DM [3]. These technology apps create portals for dietary journals, physical activity, and glucose logging and provide opportunities for scheduled prompting and reminders for medication use and other interventions [6]. Some of these technologies also allow access to information through reviews to support communication between healthcare professionals and patients. Self-management is considered one of the most effective approaches to help improve patient medication compliance, control clinical symptoms, and mitigate the adverse effects of the disease [7]. An earlier study from the Middle East reported a positive attitude of T2DM patients towards the use of mobile phones and the Internet, along with an inclination to monitor their blood glucose levels, diet planning, and contact with their doctors [8].

Approximately 1200 diabetes-related mHealth apps were available in the market in 2016, thus showing international interest in mHealth as a potential intervention solution for managing people with T2DM [9]. Indeed, several previous studies demonstrated the positive effects of mHealth apps on improving glycemic control (HbA1C), lifestyle behavior, and frequency and regularity of blood sugar measurements [10,11]. In addition, a feasibility pilot study conducted in Saudi Arabia assessed the mobile-based diabetes management and education system (SAED) and reported the significant impact of the system in reducing the HbA1C among 20 people with T2DM, which decreased from 8.76% to 7.85% (P = 0.012) [12]. However, most of these mHealth apps and systems have been developed for individual studies, and there is still a lack of supporting evidence regarding their effectiveness and usage in real-world settings.

The successful development of mHealth apps would require an adequate understanding of users’ acceptance and preference for various functionalities. Indeed, several mHealth apps for chronic diseases did not involve users in the app development process or assess usage and acceptance [13,14]. Saudi Arabia currently has about 33.55 million smartphone users, estimated to reach 36.55 million in 2028 [15,16]. However, mHealth apps to promote the self-management of chronic diseases in Saudi Arabia are in their initial stages. The key to the success of any app is to assess the targeted people’s willingness and ensure their acceptance while facilitating their use of the technology [17]. Consequently, this study investigated the acceptability and attitude toward using apps for T2DM self-management in Saudi Arabia.

## Materials and methods

### Study design, setting, and population

This cross-sectional, questionnaire-based study was conducted in the Kingdom of Saudi Arabia. Between the 1st of February and the 30th of June 2024, data were collected using an online questionnaire administered in Arabic using convenience and purposive sampling techniques. Several approaches have been used to collect data and recruit people with T2DM, including social media apps (WhatsApp and Telegram) and diabetic centers. The inclusion criteria were individuals with T2DM, those between 18 and 45 years of age who lived in Saudi Arabia, and people with another type of diabetes who were excluded from the study. The included age range was selected based on the results of a previous study [18,19] that explored medication adherence and its associated barriers among people with T2DM in Saudi Arabia and found that people with T2DM aged 45 years or younger demonstrated the highest levels of non-adherence and could benefit significantly from using mHealth apps.

### Sample size

The sample size for the current study was calculated using the Sampsize Calculator (http://sampsize.sourceforge.net/iface/). Based on a prevalence rate of 16.4%, as reported in a previous systematic review conducted in Saudi Arabia [20], along with a 95% confidence interval and precision of 5%, the estimated sample size for the current study was 210, adding 10% to allow for withdrawal from the study. Therefore, 231 patients were included in this study.

### Data collection tool

The questionnaire was developed and adapted from an existing questionnaire used in a previous study that assessed the acceptance of a self-management smartphone app among Parkinson’s patients in China [25]; however, questions related to current and future use of mHealth were added to the questionnaire. The study questionnaire comprised 29 questions divided into five sections. Section I consisted of 11 questions related to the demographic and clinical characteristics of the respondents, whereas Section II included five questions related to mobile phone usage. Section III had one question that asked about the preferred features of the T2DM self-management app, whereas Section IV included nine questions to assess the willingness to use the mHealth solution for T2DM with a self-management system. Section V had three questions to assess the attitude and intention to use the mHealth app.

Furthermore, the questionnaire was designed to include several response scales and closed-ended questions. The questionnaire draft was prepared after careful deliberation, after which it was evaluated by a three-member panel of experts to ensure its validity. The experts included a physician specializing in diabetes, a nurse with experience in diabetes care, and a social worker working with patients with diabetes. Their feedback was incorporated to refine and improve the instrument before its implementation. After the validation, the questionnaire was translated into Arabic by a professional translator. A back-translation process was conducted to verify the accuracy of the translation. This involved translating the Arabic version back into the original language by a different translator and then comparing this back-translated version with the original to ensure consistency and accuracy of meaning.

### Measures

#### Demographic and clinical characteristics.

Respondents were asked to indicate their gender, age, marital status, educational level, employment status, and place of residence. In addition, questions were asked about the duration of T2DM, presence of other chronic conditions, number of prescribed antidiabetic drugs, total number of medications used daily, and sources of T2DM information.

#### Mobile phone usage.

A total of five questions (with yes/no responses) were asked regarding ownership of cell phones (smartphones or non-smartphones), their ability to install new apps, their Internet usage habits, awareness, and previous use of mHealth apps for chronic disease management.

#### Preferred features for T2DM self-management apps.

For this question, respondents were asked to indicate their preferences pertaining to the preferred features of the app. Respondents were able to select more than one option to answer this question. The listed preferences included dietary planning, physical activity planning, medication reminders, glucose reading, recording, and tracking, as well as communication with other patients with T2DM and healthcare providers.

#### Willingness to use mHealth solutions.

Nine questions on a five-point Likert scale ranging from strongly agree to strongly disagree were used to assess willingness to use mHealth solutions. The questions were related to factors that could influence the use of mHealth apps, including cost, ease of operation, potential for quicker medication adjustments, privacy protection, reminders for following doctors’ directions, potential to reduce psychological burden, potential to reduce frequency and cost of medical consultations, improved communication with doctors and overall usefulness in T2DM management.

#### Attitude and intention to Use mHealth apps.

Respondents were asked to indicate yes or no to questions related to confidence in using smartphone apps for diabetes management, their willingness to accept help from family/friends if unable to use the app, and their future intention to use smartphone apps for T2DM management.

### Data analysis

Data were collected from the spreadsheets provided by Google Forms and transferred to Microsoft Excel and then to IBM SPSS (version 25.0) software for statistical analyses. Descriptive analysis (frequencies and percentages for categorical variables and mean±Standard Deviation (SD) for continuous variables) was performed for all variables included in the study. The correlation analysis using Pearson chi-squared and Spearman tests were performed to assess the correlation between willingness toward using the mHealth app and demographic and clinical data, and significance was considered if the p-value was less than 0.05 (5% p-value).

### Ethics approval

This study was approved by the Research Ethics Committee of Jazan University, Jazan, Saudi Arabia, in accordance with the Declaration of Helsinki. The approval number is REC-45/07/942. Participation in the current study was voluntary, and participants could withdraw from the study at any time without any potential consequences. Before completing the questionnaire, participants were informed that the data would only be used for scientific purposes and that confidentiality would be protected. Returning the completed questionnaire to the researcher was considered implied consent [22].

## Results

A total of 424 individuals with T2DM responded to and returned the questionnaire. Most respondents were female, 262 (68.8%), and lived in urban areas (215, 50.7%). The average age of the respondents was 35.86 ± 9.423, ranging from 18 to 45 years. Other key demographic data are presented in Table 1.

**Table 1:** Demographic and clinical characteristics of respondents (N = 424).

Demographics		N%/ m ± SD
**Gender**	Female	262 (68.8%)
	Male	162 (38.2%)
**Age**		35.86 Years ± 9.423 (R: 18–45)
**Marital Status**	Married	252 (59.4%)
	Single	140 (33%)
	Other (Divorced, Widowed)	32 (7.5%)
**Education Level**	Primary school	40 (9.4%)
	Middle & High school	88 (20.8%)
	Diploma	64 (15.1%)
	University	204 (48.1%)
	Postgraduate	28 (6.6%)
**Profession (Employment status)**	Employed	245 (57.8%)
	Unemployed	179 (42.2%)
**Resident Area**	Urban	215 (50.7%)
	Rural	209 (49.3%)
**Duration of T2DM condition (Years)**		6 years ± 3.5 (R: 1–12)
**Anti-Diabetic medications**	Two medications or less	215 (50.7%)
	Three medications or more	209 (49.3%)
**Co-morbidities**	Yes	200 (47.2%)
	No	224 (52.8%)
**Number of daily medications used**		3 ± 1.25 (R:2–6)

Almost all respondents mentioned owning a mobile phone, 392 (92.5%) owned a smartphone, and 32 (7.5%) used a basic mobile phone. When asked about the ability to search and download an app using a smartphone or iPad, 87.7% (372) reported the ability to do so, and 12.3% (52) did not. In addition, 89.6% (380) reported the ability to search for information using the internet and smartphones. Most of the respondents reported that they obtained diabetes-related information from their physicians 260 (61.3%), from their family members and friends 96 (22.6%), from searching the internet 48 (11.3%), and from social media (e.g., WhatsApp) 20 (4.7%).

### Preference for mHealth app functionalities for T2DM self-management

**Fig 1:**
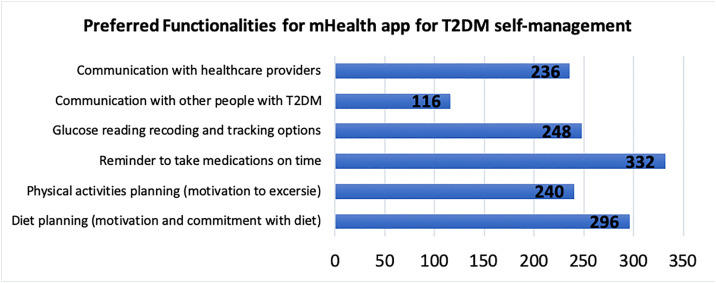
People with T2DM preferred functionalities for the mHealth app for T2DM self-management.

Most respondents, 228 (53.8%), were aware of the availability of mHealth apps intended to promote self-management of chronic diseases such as T2DM; however, only 52 out of 424 respondents reported previous experience using mHealth apps. People with T2DM reported interest in various functionalities of the mHealth app for T2DM self-management, as shown in Fig 1. Most of the respondents were interested in reminders to take medications on time, diet planning, recording and tracking blood glucose levels, planning physical activities, connecting with healthcare providers such as physicians, nurses, and pharmacists, and finally, connecting with other people with T2DM was 22.6, 20.2, 16.9, 16.3, 16.1, and 7.9%, respectively.

### Attitude and willingness toward using the mHealth app for T2DM self-management

Respondents’ attitudes toward the app were assessed to understand the elements that might impact the use of the mHealth app for T2DM self-management. Indeed, the attitudes were positive, and the majority of respondents indicated that they would use apps for T2DM self-management if they were free, easy to use, reminded them to adhere to their physician’s instructions, protected their privacy, were useful in managing T2DM, and reduced the psychological and economic burden that might be associated with T2DM (Table 2).

**Table 2:** Attitude and beliefs toward mHealth app for T2DM.

Questions	Strongly Agree	Agree	Neutral	Disagree	Strongly disagree
**I would use it if it were free**	168 (39.6%)	248 (58.5%)	8 (1.9%)		
**I would try it out if it were easy to operate**	164 (38.7%)	260 (61.3%)	_		
**I would use it if it allowed the doctor to make a medication change quicker**	116 (27.4%)	288 (67.9%)	20 (4.7%)		
**I would use it if it protected my privacy**	180 (42.5%)	244 (57.5%)	_		
**I would use it if it will help remind me to follow doctors’ directions**	168 (39.6%)	252 (59.4%)	4 (0.9%)		
**I would use it if it will reduce the psychological burden of T2DM**	148 (34.9%)	256 (60.4%)	20 (4.7%)		
**I would use it if it will reduce the frequency of seeking medical advice and the costs**	124 (29.2%)	264 (62.3%)	36 (8.5%)		
**I would use it if it will be helpful for me to communicate with the doctor.**	174 (32.5%)	362 (67.5%)	_		
**I would use it, if it will be useful in managing my T2DM disease**	111 (26.2%)	313 (73.8%)	_		

The majority of respondents, 313 (73.8%), reported confidence in their ability to use the mHealth app in the future to facilitate T2DM self-management, and 416 (98.1%) reported willingness to use it. In addition, 313 out of 424 respondents reported that they would seek assistance from family members or friends if they needed to use the future mHealth app for T2DM.

The correlation between willingness to use the mHealth app for T2DM and demographic and clinical characteristics was assessed to better understand potential users’ characteristics, as shown in Table 3. A weak positive and significant correlation was found between willingness to use the mHealth app and education level and the number of medications used daily (P = 0.02 and P = 0.04, respectively). These findings suggest that respondents with higher educational attainment and those using three or more medications per day may be more inclined to adopt and use mHealth apps. This could be due to high health literacy and the perceived value of mHealth app support in managing their condition on a daily basis. However, no significant correlation was found with age (r = –0.043, P = 0.99); this could be due to the fact that this study included only a younger population. Similarly, duration of T2DM showed a weak and non-significant positive correlation (r = 0.110, P = 0.08), suggesting a potential, but not statistically supported, trend toward increased acceptance among those with longer disease experience.

**Table 3:** Correlation analysis between respondents’ characteristics and willingness to use the mHealth app for T2DM.

	Correlation coefficient	P value
**Age**	− 0.43	0.99
**Education Level**	0.376	0.02
**Duration of T2DM (Years)**	0.110	0.08
**Number of daily medications used**	0.141	0.04

## Discussion

The principal findings of this study reveal a high level of interest and willingness among people with T2DM in Saudi Arabia to use mHealth apps for self-management. Despite widespread smartphone ownership and internet usage among respondents, only a small percentage had previously used mHealth apps for diabetes management. The study identified key desired functionalities for such apps, including medication reminders, diet planning, blood glucose tracking, and communication with healthcare providers. Importantly, respondents showed positive attitudes towards using mHealth apps, particularly if they were free, user-friendly, and offered features to enhance diabetes management and reduce associated burdens.

The World Health Organization defined mHealth as using mobile devices, such as mobile phones (smartphones), personal digital assistants, and wireless devices, to support medical and public health practices [21,23]. Currently, smartphones include features such as sensors, which can facilitate the diagnosis of chronic diseases and track and manage the patient’s condition. Several commercial mHealth apps are available, such as MySugr, Glucose Buddy, and Glucose Tracker-Diabetic Diary; however, there is no evidence of their acceptance and usage among people with T2DM. In addition, several clinical trials and feasibility studies have been conducted to assess the impact of mHealth apps on diabetes self-management, with no evidence of their effectiveness and usage in real-world practice [10,11]. Assessing mHealth app users’ acceptance and involving them in the early stages of the app design process is a key factor that could influence their adoption of these apps [22]. Therefore, this study was conducted to examine the acceptability and willingness of mHealth apps that aim to facilitate self-management among people with T2DM in Saudi Arabia. The findings of this study showed that using the mHealth app for T2DM self-management is desirable, which is unexpected, as 83.83% and 91% of people aged between 12 and 65 years use the Internet and cell phones in Saudi Arabia, respectively [23]. In this study, almost all respondents owned a cell phone, particularly a smartphone. The majority of people with T2DM (89.6% (380/424)) reported the ability to search the Internet for information; however, a few respondents (11.3% (48/424) had used it to search for information about their T2DM. However, half of the respondents were aware of the availability of self-management mHealth apps for chronic diseases (228/424), and only 52/424 reported previous and current use of such apps to manage their conditions, which is in contrast to previous studies that reported the need to improve awareness regarding self-management apps among people with cardiovascular, Parkinson’s, and diabetic diseases [24–26]. The reason for this could be the selection criteria of the participants in the current study, with individuals aged between 18–45 years included. Furthermore, several previous studies have suggested that users’ cultures, knowledge, perceptions, and experiences can significantly impact the use of mHealth apps [27,28,34,36].

In line with a previous study, people with T2DM in the current study were eager for mHealth app features that facilitated medication and dietary management, supported the monitoring and recording of blood glucose levels, and encouraged physical activity [9,29,36]. Furthermore, features that enable communication with healthcare providers and other people with T2DM reveal a paucity of communication with doctors in Saudi Arabia [30,36]. Furthermore, in consistency of other studies, the respondents indicated their willingness to use future mHealth apps to support self-management if it was free, easy to operate, proved to help remind them to take their medications on time, enabled them to communicate with their healthcare providers to obtain advice to manage better and control their conditions [26,31,34]. Therefore, mHealth app designers must consider these features in order to improve the acceptance and usage of such apps. However, interestingly, none of the participants selected “disagree” or “strongly disagree” for the questionnaire items related to the attitude and beliefs toward the mHealth app. While this may indicate a positive perception toward mHealth apps among this population, several potential response biases, especially in healthcare-related contexts, could contribute to this response pattern, such as social desirability or acquiescence biases, where respondents tend to provide favorable responses [32,33]. The lack of negative responses may also reflect participants’ optimism or high expectations regarding the benefits of mHealth apps, especially when introduced as a supportive approach to self-managing a chronic condition like T2DM. Therefore, it is important to consider these potential biases when interpreting the findings of this study, as the absence of disagreement does not necessarily imply unanimous agreement. Instead, it could highlight the need for further studies that incorporate a qualitative approach to understand perspectives better and identify potential concerns that may not be revealed through self-reported questionnaires.

Similar to previous studies, there was a significant correlation between highly educated people with T2DM, the number of medications they use daily, and their willingness to use mHealth apps [26]. However, in contrast to previous studies, no correlation was found between age, disease duration, and willingness to use mHealth apps. The reason for this could be that previous studies focused on the acceptance of the elderly population toward mHealth app use [25,26]. Finally, similar to previous studies that assessed the use of mHealth apps among diabetic populations in the Middle East region, the general attitude toward using mHealth apps was positive, and involving family members and friends was essential for encouraging and improving the acceptance of mHealth apps for T2DM self-management [34–36].

The strength of this study is the large number of completed responses, which is more than the required sample size; however, due to the online distribution method, the exact response rate could not be calculated. However, this study had several limitations that should be considered when interpreting the findings. Firstly, the cross-sectional nature of quantitative design may limit the ability to establish causal relationships or track changes in attitudes and acceptance over time. Future longitudinal studies could provide more insights into how perceptions of mHealth apps evolve with continued use. The adoption of a mixed-methods approach would also provide comprehensive insights into the preferences and attitudes of the respondents. Indeed, the research tool used in this study was a self-developed questionnaire based on validated instruments. However, it may not capture all nuances of mHealth acceptance and attitudes. Therefore, a more comprehensive validation process for the survey instrument could enhance the reliability of this study’s findings. In terms of variables studied, this study focused primarily on demographic factors and general attitudes towards mHealth, and it did not explore in-depth psychological factors, such as health beliefs or technology self-efficacy, which could influence mHealth acceptance. This study also did not assess respondents’ prior experience with other health-related mobile apps and the potential cultural factors specific to Saudi Arabia, which could have influenced their attitudes and acceptance toward the mHealth app. Finally, this study focused on assessing the general attitude and acceptance of mHealth apps for T2DM self-management without assessing the actual usage of such apps. Therefore, future studies are needed to assess the effectiveness and usage of apps in facilitating self-management among people with T2DM. The use of convenience, purposive, and self-selection sampling may have introduced bias and reduced reliability. The focus on people with T2DM aged 18–45 years limits generalizability to older populations, who make up a large proportion of T2DM patients. The sample was skewed towards those with higher education levels, which could have influenced the acceptance of the mHealth apps. Additionally, the potential for selection bias was acknowledged, as participants were recruited from a diabetes clinic and may represent a more engaged subset of people with T2DM. This could limit the generalizability of the findings to the broader T2DM population in Saudi Arabia. While the sample size was adequate for general analyses, it may have limited the ability to conduct more granular subgroup analyses or detect smaller effect sizes in some statistical comparisons. These limitations provide important directions for future research, including the need for longitudinal studies, more comprehensive assessment tools, and exploration of additional variables that may influence mHealth acceptance and use among T2DM patients in Saudi Arabia.

This study has important clinical implications for the management of T2DM in Saudi Arabia. The high acceptance and positive attitudes towards mHealth apps among people with T2DM suggest that these tools could be effectively integrated into diabetes care plans. Healthcare providers should consider recommending appropriate mHealth apps to their patients as part of a comprehensive self-management strategy. The identified preferred features, such as medication reminders, diet planning, and blood glucose tracking, can guide the development of more user-centered apps. Future research should focus on evaluating the long-term effectiveness of mHealth apps in improving clinical outcomes for T2DM patients. Studies examining the impact of these apps on glycemic control, medication adherence, and quality of life would be valuable. Additionally, investigating potential barriers to sustained app usage and exploring ways to enhance user engagement over time could provide insights for improving mHealth interventions in diabetes care.

## Conclusion

This study helped understand the acceptance and attitude toward self-management of mHealth apps among people with T2DM in Saudi Arabia. Indeed, most people with T2DM in Saudi Arabia own smartphones and express positive attitudes toward using mHealth apps to facilitate self-management of their condition. Moreover, this study found that mHealth apps for T2DM need to be free, have a simple interface, support diagnosis, manage medications, monitor disease conditions, motivate lifestyle changes, support communication with healthcare providers, and reduce the psychological burden of these conditions. This could provide important implications for future app design based on the end-user’s most necessary features and functionalities.

## Supporting information

S1 File Exploring the acceptability of smartphone applications to aid self-management for patients with type 2 Diabetes Mellitus: A descriptive cross-sectional study. (PDF)
