# Supplementary material for: Exploring the acceptability and attitude toward mobile health applications to aid self-management for people with type 2 diabetes mellitus in Saudi Arabia: A descriptive cross-sectional study
Source: PLoS One. 2025 Nov 26;20(11):e0331314. doi: 10.1371/journal.pone.0331314 (PMC12654941; doi:10.1371/journal.pone.0331314)
Supplement: S1 File — (PDF) [file pone.0331314.s001.pdf]

Research Tool-Questionnaire

Title: Exploring the acceptability of smartphone applications to aid self-management for patients with type 2 Diabetes Mellitus: A descriptive cross-sectional study.

Greetings! You are invited to participate in a study being conducted by Dr. Amani Khardali (Assistant Professor, Department of Clinical Pharmacy, College of Pharmacy, Jazan University).

The information collected in this survey will be used as part of a research study. There will not be any personally identifiable information collected in this survey, and all responses will be kept in the strictest confidence. Prior to analyzing the data, the order of respondents will be randomized to ensure your responses remain anonymous.

Your participation in this survey is voluntary. You may stop your participation in this survey at any time if you want.

The purpose of this study is to explore the acceptance and attitude of T2DM patients towards the use of smartphone applications to aid the self-management of their condition.

If you have any questions or concerns regarding the study, please do not hesitate to contact me at aakherdeli@jazanu.edu.sa

PLEASE ANSWER THE QUESTIONS BELOW BEFORE PROCEEDING TO THE QUESTIONNAIRE. Please indicate by checking the box below that you have read and agree to the above information.

□ I have read and understand the above information and AGREE to participate in this research study.

□ I have read and understand the above information and DO NOT AGREE to participate in this research study.

Press Next:

Section I:
Demographic and clinical characteristics of participants:

1. Gender: □ Male □ Female
2. Age： __________ years
3. Marital Status: □ Unmarried □ Married. □ Other (Divorced, Widowed)
4. Education Level：□Primary school □ Middle school or High school
   □Diploma □ University □ postgraduate
5. Employment status: □ Employed □ Unemployed
6. Place of Resident: □Rural □Urban

<u>Clinical:</u>

7. T2DM Duration of diagnoses： ___________________ years.
8. Do you have any other chronic condition? □ Yes (Name of disease….) □ No
9. Number of anti-diabetic drugs: □ ≤two drugs □≥three drugs
10. Number of medications used per day:……….
11. Source for obtaining T2DM disease information □Doctor (Outpatient) □ Computer/ internet □Cell phone □ Social media □ Friends and family member

Section II:

Mobile phone usage:

1. Do you have a cell phone? □Yes □No

If yes, Do You have □non-smartphone □ Smartphone?

2. Are you able to install a new app on your phone or tablet?

□ Yes □ No

3. Do you usually search the Internet? □Yes □No

5. Have you ever heard of a mobile self-management application to aid the use of medication for chronic diseases? (Such as managing diabetes and high blood pressure ） □Yes □No

5.5. If Yes, have you previously used the mHealth app to manage your condition? □Yes □No

Section III:

1. If you want to use a smartphone application to help self-manage T2DM, select what management services/functionalities you would be interested in (Select all apply):

Preferred features
o Dietary planning
o Physical Activity Planning
o Reminders to take medication on time
o Glucose reading recoding and tracking options
o Communication with other patients with T2DM
o Communication with Healthcare providers, such as physicians, dieticians, nurses, and pharmacists.

Section IV:

Willingness to use mHealth solution with a self-management system.

7. I would use it if it were free:

□Strongly agree □Agree □Neutral □Strongly disagree □Disagree

8. I would try it out if it were easy to operate:

□Strongly agree □Agree □Neutral □Strongly disagree □Disagree

9. I would use it if it allowed the doctor to make a medication change quicker:

□Strongly agree □Agree □Neutral □Strongly disagree □Disagree

10. I would use it if it protected my privacy:

□Strongly agree □Agree □Neutral □Strongly disagree □Disagree

11. I would use it if it will help remind me to follow doctors' directions:

□Strongly agree □Agree □Neutral □Strongly disagree □Disagree

12. I would use it if it will reduce the psychological burden of T2DM:

□Strongly agree □Agree □Neutral □Strongly disagree □Disagree

13. I would use it if it will reduce the frequency of seeking medical advice and the costs:

□Strongly agree □Agree □Neutral □Strongly disagree □Disagree

14. I would use it if it will be helpful for me to communicate with the doctor:

□Strongly agree □Agree □Neutral □Strongly disagree □Disagree

15. I would use it if it will be useful to manage my T2DM disease:

□Strongly agree □Agree □Neutral □Strongly disagree □Disagree

Section V:

Attitude and intention to use the mHealth app:

16. I am confident that I could use a smartphone application to help me with my diabetes.

□ Yes □No

17. If you were unable to use a smartphone app, would you want a spouse/friend/ family member to help you manage your diabetes with the app?

□ Yes □ No

18. I intend to use a smartphone application to support me with my T2DM management in the future.

□ Yes. □ No

Thank you for your interest in completing this questionnaire
